# Rac1 Regulates the NLRP3 Inflammasome Which Mediates IL-1beta Production in *Chlamydophila pneumoniae* Infected Human Mononuclear Cells

**DOI:** 10.1371/journal.pone.0030379

**Published:** 2012-01-20

**Authors:** Julia Eitel, Karolin Meixenberger, Claudia van Laak, Christine Orlovski, Andreas Hocke, Bernd Schmeck, Stefan Hippenstiel, Philippe Dje N'Guessan, Norbert Suttorp, Bastian Opitz

**Affiliations:** Department of Internal Medicine, Infectious Diseases and Pulmonary Medicine, Charité – Universitätsmedizin Berlin, Berlin, Germany; University of California Merced, United States of America

## Abstract

*Chlamydophila pneumoniae* causes acute respiratory tract infections and has been associated with development of asthma and atherosclerosis. The production of IL-1β, a key mediator of acute and chronic inflammation, is regulated on a transcriptional level and additionally on a posttranslational level by inflammasomes. In the present study we show that *C. pneumoniae*-infected human mononuclear cells produce IL-1β protein depending on an inflammasome consisting of NLRP3, the adapter protein ASC and caspase-1. We further found that the small GTPase Rac1 is activated in *C. pneumoniae*-infected cells. Importantly, studies with specific inhibitors as well as siRNA show that Rac1 regulates inflammasome activation in *C. pneumoniae*-infected cells. In conclusion, *C. pneumoniae* infection of mononuclear cells stimulates IL-1β production dependent on a NLRP3 inflammasome-mediated processing of proIL-1β which is controlled by Rac1.

## Introduction

*Chlamydophila pneumoniae* is a gram-negative, obligate intracellular bacterium that causes acute respiratory tract infections including pneumonia, sinusitis, and bronchitis. Moreover, chronic or recurrent *C. pneumoniae* infections have been associated with development of chronic lung diseases such as asthma [1] as well as with development of vascular lesions and atherosclerosis [2]–[5]. *Chlamydiae* undergo a biphasic developmental cycle inside an internalized vesicle termed inclusion. The virulent and metabolically inert elementary bodies (EBs) differentiate into the non-virulent and metabolically active reticulate bodies (RBs), re-differentiate to EBs and finally escape from the host cell. *C. pneumoniae* is able to reside and replicate in different cell types such as monocytes, macrophages, smooth muscle cells and endothelial cells, and often persists intracellularly for indefinite periods [6].

The innate immune system senses microbial pathogens by pattern recognition receptors (PRRs) including the membrane-bound Toll-like receptors (TLRs) and C-type lectin receptors (CLRs), as well as the cytosolic RIG-like receptors (RLRs) and NOD-like receptors (NLRs) [7]–[9]. The TLRs, RLRs as well as some NLRs like NOD1/NLRC1 and NOD2/NLRC2 activate a NF-κB-dependent expression of proinflammatory genes including, for example, TNFα and proIL-1β. In contrast, other NLRs like NLRC4 (also called IPAF), NLRP1 and NLRP3 are involved in the assembly of a multiprotein complex called inflammasome, which also contains the adaptor molecule ASC (apoptosis associated speck-like protein containing a caspase activation recruitment domain) and caspase-1. Inflammasomes mediate caspase-1 activation leading to cleavage of proIL-1β and proIL-18 into their active forms [10]. Inflammasomes are activated by a variety of pathogens and endogenous danger signals. The NLRP3 inflammasome, for example, is stimulated by bacteria expressing pore-forming toxins or secretion systems [11]–[19], uric acid crystals, ATP, silica crystals and aluminium salts [16], [20]–[22]. While it is unlikely that these different stimuli directly bind to NLRP3, the precise signal that is detected by NLRP3 has not been unambiguously identified.

The Rho family GTPase Rac1 is a key regulator of various cellular functions such as cytoskeletal reorganization or cellular growth [23]–[25]. It is also implicated in different aspects of antibacterial host defense, including leukocyte chemotaxis [24], pathogen phagocytosis [26], [27], ROS production [28], and regulation of TLRs and NOD2 [29]–[32].

*Chlamydia* infection is detected by TLR2 and TLR4 [33]–[36] at the plasma membrane, and by NOD1 and NOD2 in the cytosol [37]–[40] which leads to stimulation of pro-inflammatory gene expression. Additionally, a yet-to-be-identified cytosolic PRR activates type I IFN production upon sensing of *Chlamydia* species [41], [42].

In this study, we demonstrate that the NLRP3 inflammasome regulates IL-1β production in *C. pneumoniae*-infected human monocytes. The data further show that the small GTPase Rac1 controls the *C. pneumoniae*-stimulated inflammasome activation.

## Methods

### Ethics statement

Studies with human PBMCs were approved by the ethics committee of the Charité-Universitätsmedizin Berlin. Written informed consent from all participants was obtained. This study was carried out in strict accordance with the recommendations in the Guide for the Care and Use of Laboratory Animals of the Committee of animal welfare commissioners in Berlin, Germany. The protocol was approved by the Committee on the Ethics of Animal Experiments of the Charité-Universitätsmedizin Berlin and the governmental institutions (LAGeSo, Permit Number: T 0013/11).

### Bacterial strains

The *C. pneumoniae* strain CWL029 (ATCC VR-1310) was cultured and purified in HEp-2 cells as described by Maass et al. [3]. Isolated PBMCs or cultured THP-1 cells and BMMs were inoculated with *C. pneumoniae* using a multiplicity of infection (MOI) of 0.5 to 5. Plates were centrifuged at 800 *g* at 37°C for 1 hour and subsequently incubated for different time intervals.

### Human cell culture

The human monocyte cell line THP-1 was obtained from DSMZ. Cells were cultured in RPMI 1640 containing 5% FCS (Gibco) and seeded in appropriate culture dishes at a concentration of 10^6^ cells/ml. THP-1 were left untreated or adhered o/n with 100 ng/ml PMA (Sigma-Aldrich) and cultured two more days before infection. Human PBMCs were isolated from buffy coat preparations obtained from the German Red Cross Berlin. The buffy coat was diluted with RPMI 1640 containing 5% FCS and 0.2 mM EDTA, and centrifuged twice over Pancoll (PAN Biotech) for 25 min at 800× g. Cells were cultured in RPMI 1640 containing 10% FCS and seeded in appropriate culture dishes at a concentration of 5*10^6^ cells/ml. In some experiments, human PBMCs or THP-1 cells were preincubated for 30 min with 25 or 50 µM NSC23766 (Calbiochem, San Diego, CA), or substances were added 2.5 h post-infection.

### Generation of mouse BMMs

The protocol was approved by the Committee on the Ethics of Animal Experiments of the Charité-Universitätsmedizin Berlin and the governmental institutions (LAGeSo, Permit Number: T 0013/11). Wildtype C57BL/6 mice were purchased from Charles River. Nlrp3 knockout mice were kindly provided by Professor Jürg Tschopp, University of Lausanne [22]. Bone marrow-derived macrophages (BMMs) were cultured in RPMI 1640 containing 30% L cell supernatant and 20% FBS, and were replated one day prior to infection in RPMI 1640 containing 15% L cell supernatant and 10% FBS.

### RNA interference

THP-1 cells were transfected using the Amaxa Nucleofector (Amaxa) according to the manufacturer's protocol (Cell Line Nucleofector Kit V, Program T-08) with 2 µg per 10^6^ cells. Human PBMCs were transfected using the Amaxa Nucleofector according to the manufacturer's protocol (Human Monocyte Nucleofector Kit, Program Y-01) with 4 µg siRNA per 10^7^ cells [30]. Control non-silencing small interfering RNA (siRNA) (sense UUCUCCGAACGUGUCACGUtt, antisense ACGUGACACGUUCGGAGGAGAAtt), and siRNAs targeting ASC (sense GAUGCGGAAGCUCUUCAGU, antisense ACUGAAGAGCUUCCGCAUC), NLRP3 (sense GGUGUUGGAAUUAGACAAC, antisense GUUGUCUAAUUCCAACACC), and caspase-1 (sense GGUUCGAUUUUCAUUUGAG, antisense CUCAAAUGAAAAUCGAACC) were purchased from Ambion (Austin, TX). Rac1 (sense CACCACUGUCCCAACACUC, antisense GAGUGUUGGGACAGUGGUG) were purchased from MWG-Biotech AG.

### Western Blot

Cell-free supernatants were concentrated using Microcon Ultracel YM-3 Centrifugal Filter Devices (Millipore). Cell extracts or concentrated supernatants were separated by SDS-PAGE and blotted. Membranes were first exposed to antibodies specific for IL-1β (Cell Signaling Technology, Beverly, MA), Caspase-1 (Cell Signaling Technology) or Rac1 (BD Transduction Laboratories), and subsequently incubated with secondary antibodies (HRP-labelled anti-rabbit (Santa Cruz Biotechnology Santa Cruz, CA) or Cy5.5-labelled anti-mouse). Proteins were detected by Chemiluminescence (Pierce, USA) or using the Odyssey Infrared Imaging System (LI-COR).

### ELISA

Concentrations of IL-1β or mIL-1β in cell-free supernatants were quantified by commercially available sandwich ELISA Kits (BD Biosciences, eBioscience).

### PCR Analysis

Total RNA from THP-1 or human PBMCs was isolated using RNeasy Kit (Qiagen) and reverse transcribed with High Capacity cDNA Reverse Transcription Kit (Applied Biosystems). The generated cDNA was amplified by semi-quantitative PCR using REDTaq DNA Polymerase (Sigma-Aldrich) and specific primers, or by quantitative PCR using TaqMan Gene Expression Assays (Applied Biosystems) on the 7300 Real Time PCR System (Applied Biosystems). The mRNA expression level of each target gene was normalized to the expression level of GAPDH. For measurements of chlamydial 16s rRNA, total RNA was isolated with the RNeasy Mini kit (Qiagen). The extracted RNA was treated with DNase I (Qiagen) to eliminate the contaminating eukaryotic DNA. After reverse transcription, relative quantification of *C. pneumoniae* 16S rRNA (accession number U68426, www.ebi.ac.uk/embl; sense, 5′-ATGTGGATGGTCTCAACCCCAT-3′; antisense, 5′-GGCGCCTCTCTCCTATAAATAGG-3′) was carried out in a real-time PCR, using SYBR-Green PCR Master-Mix (Applied Biosystems, Foster City, CA) and normalized to GAPDH of the same samples.

### Confocal laser scanning microscopy

THP-1 cells were seeded on coverslips and left untreated or were infected with *C. pneumoniae* (MOI 2) in the presence or absence of NSC23766. After 20 h, cells were fixed with 3% paraformaldehyde (Sigma-Aldrich). Cells were permeabilized with 1% Triton X-100 (Sigma-Aldrich) and blocked with 5% goat serum (Invitrogen). *Chlamydia* and ASC were stained by exposing cells to specific Abs (Dako Cytomation, Cambridgeshire and Santa Cruz Biotechnology), followed by incubation with AF546-conjugated anti-mouse IgG and AF488-conjugated anti-rabbit IgG, respectively (Invitrogen). Confocal laser scanning microscopy was conducted on a LSM5 Pascal (Zeiss).

### *C. pneumonia* quantification

For HEp-2 reinfection assays, THP-1 cells transfected with control (c-siRNA) or Rac1 siRNA were exposed to *C. pneumoniae* (MOI 0.5). After 72 h, cells were washed with PBS, scraped off into medium, and rigorously vortexed with glass beads to release bacteria from the cells. Whole lysates were centrifuged at 500× g to pellet cellular debris and passaged onto HEp-2 cells seeded on glass coverslips. At 48 h postinfection (p.i.), cells were fixed and immunolabeled with anti–Chlamydia-LPS Ab as described above. Cells were analyzed using a Pascal 5 confocal laser scanning microscope (Zeiss, Jena, Germany) and *C. pneumoniae* inclusions per well were counted.

### Statistical analysis

Effects of siRNAs were statistically evaluated by a one-way ANOVA and compared by a Newman-Keul's post-test. Throughout the figures, *p*<0.05 is indicated by one asterisk and *p*<0.01 is indicated by double asterisks.

## Results

### *C. pneumoniae* stimulates production of mature IL-1β in human monocytic cells

**Figure 1:**
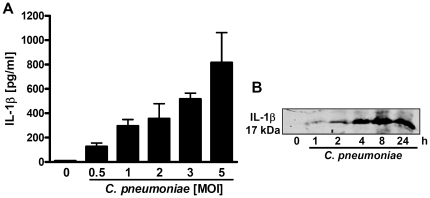
*C. pneumoniae* stimulates production of mature IL-1β in human PBMCs. (A) Human PBMCs were infected with different MOI of *C. pneumoniae* for 16 hrs and production of IL-1β was determined by ELISA. (B) Human PBMCs were infected with *C. pneumoniae* (MOI 3) for different time intervals and amounts of mature IL-1β (17 kDA) in the cell supernatant was visualized by Western Blot. The western blot is representative of three independent experiments. Results obtained from ELISAs represent mean ± SD of three independent experiments.

First, we infected peripheral blood mononuclear cells (PBMCs) and THP-1 cells with different MOIs (multiplicity of infection) of *C. pneumoniae* strain CWL029. *Chlamydia* infection dose-dependently stimulated IL-1β production in both cell types (Fig. 1A and data not shown). Furthermore, PBMCs were infected with *C. pneumoniae* (MOI 5) at different time points and accumulation of mature IL-1β in the cell supernatant was demonstrated by Western blot (Fig. 1B).

### The *C. pneumoniae*-induced IL-1β secretion depended on the NLRP3 inflammasome

**Figure 2:**
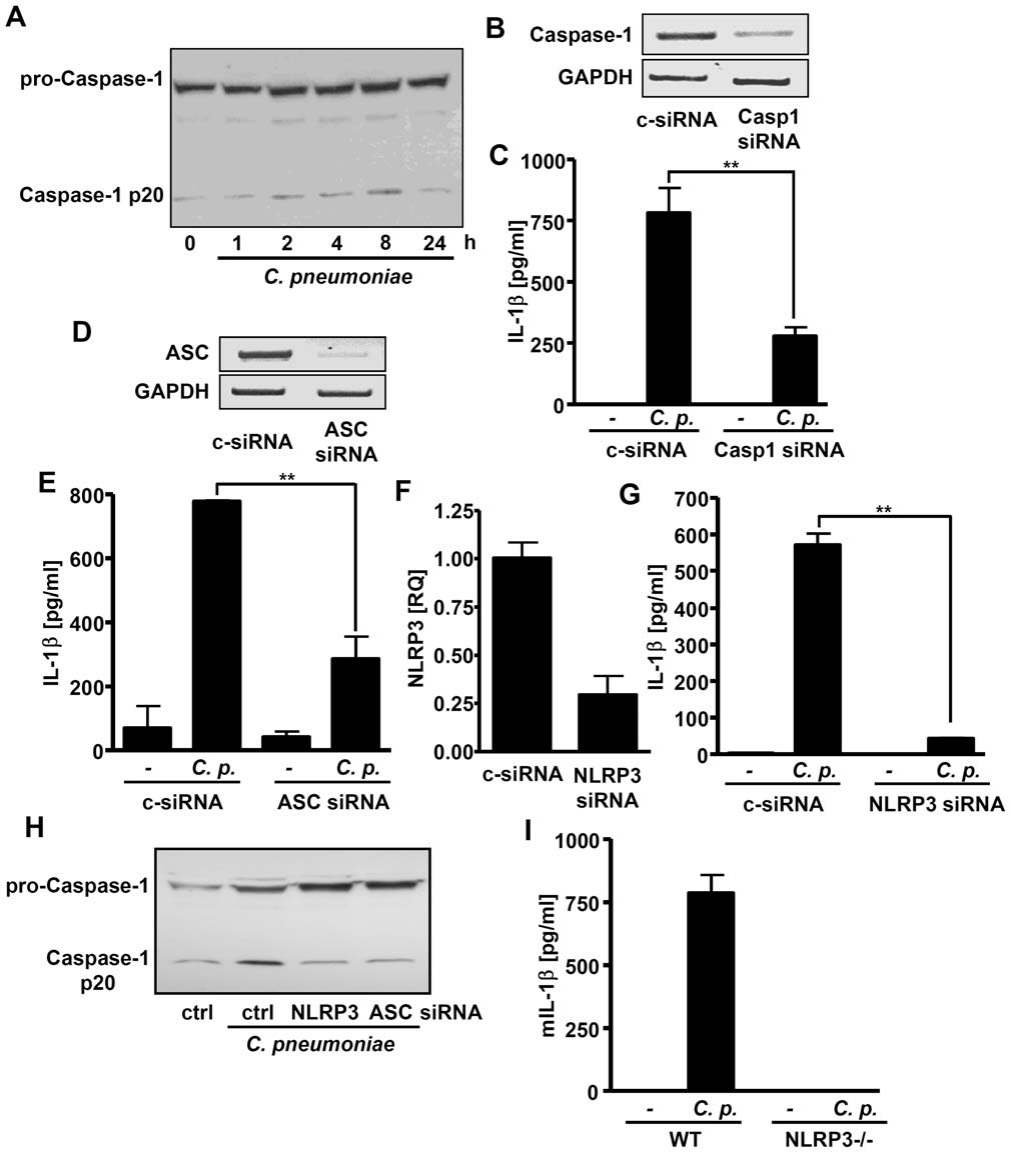
Caspase-1, ASC and NLRP3 mediate IL-1β production in *C. pneumoniae*-infected cells. (A) THP-1 monocytes were infected with *C. pneumoniae* (MOI 3) for different time intervalls. Cell lysates were harvested and assayed for procaspase-1 and caspase-1 p20. (B, C) PBMCs were transfected with control siRNA or siRNA specific for caspase-1. After 48 h, cells were infected with *C. pneumoniae* (MOI 3) for 16 hrs. Expression of caspase-1 was examined by reverse transcription PCR, and supernatants were subjected to IL-1β ELISA. (D–G) PBMCs were transfected with control siRNA or siRNA specific for ASC (D, E) or NLRP3 (F, G). After 48 h, cells were infected with *C. pneumoniae* (MOI 3), expression of ASC (D) and NLRP3 (F) was examined by reverse transcription PCR, and supernatants were subjected to IL-1β ELISA (E, G). (H) Cells were transfected with siRNA as indicated and, after 48 h, infected with *C. pneumoniae* (MOI 3). Cell lysates were assayed for procaspase-1 and caspase-1 p20. (I) Mouse BMMs obtained from wildtype and Nlrp3−/− mice were infected with *C. pneumoniae* (MOI 3) for 16 hrs. Production of mIL-1β was quantified by ELISA. Western Blots are representative for at least three independent experiments. Results obtained from ELISAs represent mean ± SD of three independent experiments.

The primary function of inflammasomes is to convert inactive pro-caspase-1 into the active, cleaved enzyme which subsequently processes proIL-1β into IL-1β. *C. pneumoniae* infection leads to cleavage and therefore activation of caspase-1 (Fig. 2A). We verified the role of caspase-1 activation for *C. pneumoniae*-mediated IL-1β release using siRNA specific for caspase-1. Knockdown of caspase-1 in PBMCs (Fig. 2B) clearly reduced the *Chlamydia*-induced secretion of IL-1β (Fig. 2C). Moreover, ASC and NLRP3 siRNAs strongly attenuated expression of their corresponding transcripts, and lowered the IL-1β production in *C. pneumoniae*-infected cells (Fig. 2D,E, F, G). ASC and NLRP3 siRNAs also inhibited activation of caspase-1 (Fig. 2H). In order to verify the involvement of NLRP3 in *C. pneumoniae*-induced secretion of IL-1β, we infected bone-marrow macrophages (BMMs) from wildtype and Nlrp3 knockout mice with *C. pneumoniae*. Our results showed that upon infection wildtype BMMs produced IL-1β whereas IL-1β production in the Nlrp3 knockout macrophages was completely abolished (Fig. 2I). These results collectively show that the NLRP3 inflammasome regulates IL-1β production at the level of caspase-1 in *C. pneumoniae*-infected host cells.

### Rac1 is essential for *C. pneumoniae*-induced inflammasome activation and IL-1β production in human monocytes

**Figure 3:**
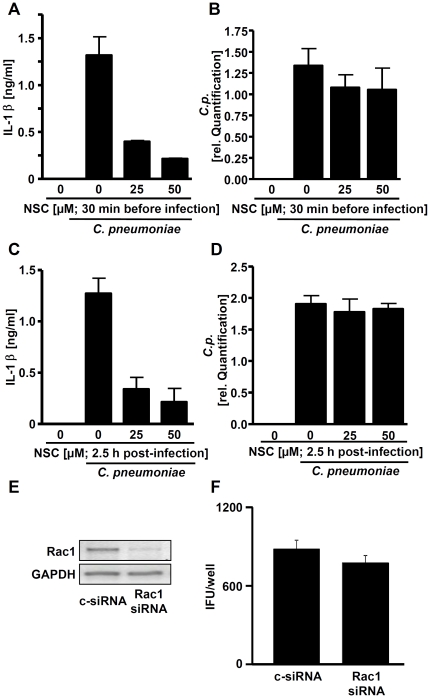
Role of Rac1 in the production of IL-1β in *C. pneumoniae*-infected cells. (A) PBMCs were incubated with different concentrations of the Rac1 inhibitor NSC23766 for 30 min and subsequently infected with *C. pneumoniae* (MOI 3), or (C) cells were first infected with *C. pneumoniae* (MOI 3) and NSC23766 was added 2.5 h post-infection. After incubating 16 hrs production of IL-1β was quantified by ELISA. Total RNA was harvested for quantification of chlamydial 16s rRNA production (B, D) using real-time PCR as indicated in the Materials and Methods section. (E) THP-1 cells were transfected with control siRNA or siRNA specific for Rac1. After 48 h, cells were infected with *C. pneumoniae* (MOI 3) for 16 hrs and knock down of Rac1 was assessed by reverse transcription PCR. (F) HEp-2 reinfection assay in which siRNA-transfected THP-1 cells infected with *C. pneumoniae* (MOI 0.5; 72 h) were harvested and inoculated onto monolayers of HEp-2 cells. Infected cells were then stained for Chlamydia 48 h p.i. and clamydial inclusions were counted. Data shown are representative for at least three (A–D) or two (E, F) experiments performed in duplicates.

Since Rac1 has been implicated in the regulation of different innate immune pathways, we analysed the role of Rac1 in the *C. pneumoniae*-stimulated IL-1β production. We preincubated PBMCs with the specific Rac1 inhibitor NSC23766 and subsequently infected them with *C. pneumoniae* at a MOI of 3. Inhibition of Rac1 decreased IL-1β release in a dose-dependent manner (Fig. 3A). Moreover, the Rac1 inhibitor also reduced the IL-1β production in *C. pneumoniae*–infected cells when added 2.5 hrs post infection (Fig. 3C). In order to rule out the possibility that reduced IL-1β production was a result of diminished bacterial load in the cells, we measured the chlamydial 16s rRNA in all samples via Q-PCR. The data show that inhibition of Rac1 did not influence bacterial numbers in the cells (Fig. 3B, D). Moreover, reinfection of HEp-2 cells, with lysates of THP-1 cells transfected with Rac1 siRNA and subsequently infected with *C. pneumoniae*, also demonstrated a similar number of inclusions compared with control siRNA treated cells (Fig. 3E, F).

**Figure 4:**
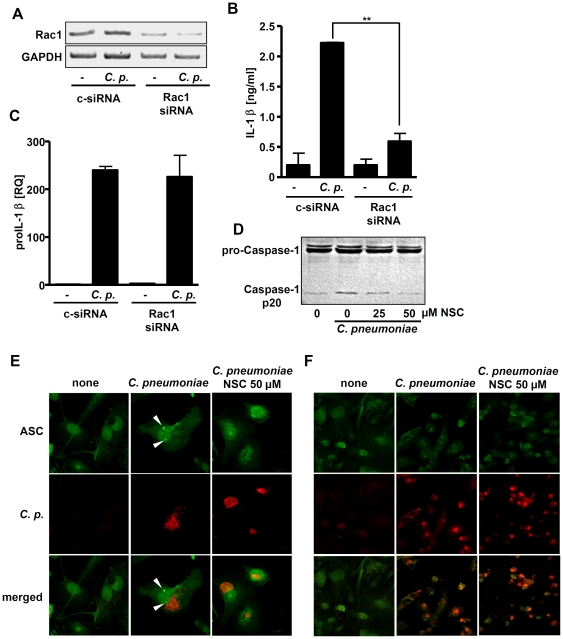
Rac1 controls IL-1β production at a posttranscriptional level in *C. pneumoniae*-infected cells. PBMCs were transfected with control siRNA or siRNA specific for Rac1. After 48 h, cells were infected with *C. pneumoniae* (MOI 3) for 16 hrs and knock down of Rac1 was assessed by reverse transcription PCR (A). Cell supernatants were subjected to IL-1β ELISA (B), and levels of pro-IL-1β mRNA were analyzed by Q-PCR (C). (D) THP-1 cells were incubated with the indicated concentrations of NSC23766 for 30 min and afterwards infected with *C. pneumoniae* (MOI 3) for 8 h. Cell lysates were assayed for pro-caspase-1 and caspase-1 p20 by Western blot. The western Blot is representative of three independent experiments. (E, F) THP-1 cells seeded on coverslips were treated or not treated with NSC23766, and infected with *C. pneumoniae* for 20 h. Bacteria (red) and ASC (green) were visualized by confocal laser scanning microscopy using specific antibodies. The arrowheads point to ASC foci. Images are representative of three independent experiments (original magnification 63×).

To substantiate our findings, we transfected PBMCs with control or Rac1-specific siRNA and infected them with *C. pneumoniae*. We found that Rac1 siRNA strongly diminished Rac1 mRNA expression (Fig. 4A). Rac1 siRNA clearly reduced *C. pneumoniae*-mediated IL-1β secretion (Fig. 4B), but did not affect induction of proIL-1β mRNA (Fig. 4C). Together, the results show that Rac1 regulates the *C. pneumoniae*-stimulated IL-1β production at a posttranscriptional level, but did not influence bacterial uptake in the cells examined.

To determine if Rac1 has an influence on the activation of caspase-1, we incubated the cells with the Rac1 inhibitor NSC23766, infected them with *C. pneumoniae* and analysed caspase-1 activation in cell lysates. As shown in Figure 4D, NSC23766 dose-dependently inhibited the *C. pneumoniae*-stimulated activation of caspase-1. Recent studies showed that in cells stimulated with inflammasome agonists or infected with different pathogens, ASC relocalized into distinct foci [43], [44]. We therefore investigated whether similar foci-like structures could be detected in *C. pneumoniae*-infected THP-1 cells. Immunofluorescence microscopy revealed that *C. pneumoniae* infection triggered the assembly of ASC foci (Fig. 4E, F). Interestingly, Rac1 inhibition by NSC23766 reduced the relocalization of ASC into distinct foci (Fig. 4E, F). Collectively, the results suggest that Rac1 regulates inflammasome activation in *C. pneumoniae*-infected monocytes upstream of ASC and caspase-1.

## Discussion

IL-1β is a key mediator of acute and chronic infections, and is therefore tightly controlled at different levels. This ensures that IL-1β is not inappropriately released, since this could have deleterious consequences for the host. We examined the posttranslational regulation of IL-1β production by inflammasomes in *C. pneumophila*-infected cells. Our data show that the NLRP3 inflammasome mediates the processing of proIL-1β into IL-1β. Importantly, we provide evidence for a regulation of the NLRP3 inflammasome by the small GTPase Rac1.

It has been shown that *Chlamydia*-infected monocytes and macrophages secrete IL-1β and this process requires caspase-1 [45]–[47]. Our results add to this by showing that the *C. pneumoniae*-stimulated IL-1β production in human PBMCs and murine BMMs was mediated by an inflammasome composed of NLRP3, ASC and caspase-1. This is in line with recent studies showing that infection of cells with *C. trachomatis* and *C. pneumoniae* led to activation of caspase-1 depending on NLRP3 and ASC inflammasome [48]–[50]. We further showed that upon infection with *C. pneumoniae* ASC foci formation occurs, which is consistent with a recent study demonstrating that infection with *S. typhimurium* or *F. novicida* causes ASC relocalization in distinct foci [43]. These foci have been suggested to represent the sites of IL-1β and IL-18 processing [43].

Our study has characterizes a mechanism of inflammasome regulation. We found that Rac1 was activated in *C. pneumoniae*-infected monocytic cells, and that ASC loci formation, caspase-1 activation and production of IL-1β in these cells were dependent on Rac1. Rac1 was not required for expression of proIL-1β. Several pathogens engage both Rho GTPases and caspase-1 to influence the host [51], [52]. *Yersinia* bacteria, for instance, can prevent host caspase-1 activation and IL-1β release by targeting Rac1 with type III effector proteins [53]. On the other hand, the *Salmonella enterica* serovar Typhimurium type III effector protein SopE activates Rac1 and thus leads to caspase-1 activation and subsequent maturation and secretion of IL-1β [54]. A Rac1-dependent regulation of inflammasomes might therefore be a common phenomenon in cells infected with pathogenic bacteria. However, the signalling pathway connecting Rac1 and caspase-1 is not understood. Rac1 might regulate NLRP3 inflammasome activation via controlling production of reactive oxygen species [55]. Moreover, the Rac1 interaction partner LIM kinase has been proposed to signal to caspase-1 [53]. Also, an interaction between the Rac1 effector “p21 activated kinase 1” and caspase-1 has been suggested [56]. Further studies examining the links between Rac1 and inflammasomes are required to fully understand their interaction in bacterial infections.

Taken together, the IL-1β production in *C. pneumoniae*-infected monocytic cells relies on the NLRP3 inflammasome. Our finding that Rac1 controls the inflammasome activation adds to the understanding of this important innate immune pathway.
